# A Class-Information-Based Penalized Matrix Decomposition for Identifying Plants Core Genes Responding to Abiotic Stresses

**DOI:** 10.1371/journal.pone.0106097

**Published:** 2014-09-02

**Authors:** Jin-Xing Liu, Jian Liu, Ying-Lian Gao, Jian-Xun Mi, Chun-Xia Ma, Dong Wang

**Affiliations:** 1 School of Information Science and Engineering, Qufu Normal University, Rizhao, Shandong, China; 2 School of Communication, Qufu Normal University, Rizhao, Shandong, China; 3 Library of Qufu Normal University, Qufu Normal University, Rizhao, Shandong, China; 4 Bio-Computing Research Center, Shenzhen Graduate School, Harbin Institute of Technology, Shenzhen, Guangdong, China; 5 College of Computer Science and Technology, Chongqing University of Posts and Telecommunications, Chongqing, China; 6 Chongqing Key Laboratory of Computational Intelligence, Chongqing University of Posts and Telecommunications, Chongqing, China; The University of Hong Kong, Hong Kong

## Abstract

In terms of making genes expression data more interpretable and comprehensible, there exists a significant superiority on sparse methods. Many sparse methods, such as penalized matrix decomposition (PMD) and sparse principal component analysis (SPCA), have been applied to extract plants core genes. Supervised algorithms, especially the support vector machine-recursive feature elimination (SVM-RFE) method, always have good performance in gene selection. In this paper, we draw into class information via the total scatter matrix and put forward a class-information-based penalized matrix decomposition (CIPMD) method to improve the gene identification performance of PMD-based method. Firstly, the total scatter matrix is obtained based on different samples of the gene expression data. Secondly, a new data matrix is constructed by decomposing the total scatter matrix. Thirdly, the new data matrix is decomposed by PMD to obtain the sparse eigensamples. Finally, the core genes are identified according to the nonzero entries in eigensamples. The results on simulation data show that CIPMD method can reach higher identification accuracies than the conventional gene identification methods. Moreover, the results on real gene expression data demonstrate that CIPMD method can identify more core genes closely related to the abiotic stresses than the other methods.

## Introduction

The changing environmental conditions have a significant impact on the survival and growth of plants. A series of various abiotic stresses can bring about the overproduction of reactive oxygen species in plants, which may damage carbohydrates, proteins, lipids and DNA resulting in oxidative stress [1]. In order to cope with these abiotic stresses, including cold, drought, heat, osmotic press, salt, UV-B light stresses, etc., plants have their own defense mechanisms to adapt the complex and changeful environment [2], [3]. In other words, a particular set of interacting plants genes which are always called core genes exist in responding to each abiotic stress. Hence, how to extract these core genes is becoming a very meaningful issue in plant science.

With the development of science and technology, the emergence of gene microarray technology [4], [5] makes it possible for researchers to monitor gene expression levels on a genomic scale [6], [7]. This not only brings us more opportunities but also more challenges to study the gene expression data. Although the DNA microarray technology allows researchers to measure the expression levels of thousands (even more than 10,000) of genes in an experiment simultaneously, the gene expression data also have the problem: the characteristic genes which biologists are interested in occupy a very small part of the whole genes. It is difficult for us to catch the small but important part of the whole genes due to the complexity and multidimensionality of the gene expression data. Therefore, it becomes an urgent issue how to identify the characteristic genes from gene expression data in an effective way.

Among a variety of methods, feature selection is demonstrated to be a simple and effective method. To obtain the features of gene expression data, feature selection methods firstly calculate a score for each feature, then choose the features which gain high scores [8]. These methods can achieve a satisfactory performance and have a significant superiority on explaining the gene expression data more intuitive. But there exists a shortcoming that feature selection methods neglect the dependencies among features since they only calculate the score for each feature respectively. The appearance of feature extraction methods can overcome the shortcoming in an effective way [9]. As a tool to reduce the dimension, feature extraction methods take all the gene expression information simultaneously into consideration to extract the genes instead of feature selection methods. Until now, singular value decomposition (SVD) and principal component analysis (PCA) are commonly used methods of feature extraction. For example Kumar et al. applied SVD on Tuberculosis and Hypertension datasets to mine association in health care data [10]. Aradhya et al. used SVD for biclustering gene expression data [11]. PCA was used to cluster gene expression data by Yeung et al. [12]. PCA was used to select genes for microarray data analysis by Wang et al. [13]. Ma et al. applied PCA for identifying differential gene pathways [14].

Although SVD and PCA have already been used to analyze the gene expression data successfully, they still have some defects. For instance, SVD's left singular vectors and right singular vectors are always dense. In the same way, this drawback exists in the principal components (PCs) of PCA. Thus, it is difficult to explain these singular vectors and PCs objectively. Researchers have proposed a variety of mathematical methods to reduce the complexity of the data and make them more intelligible and interpretable. For example Liu et al. proposed robust PCA for discovering differentially expressed genes [15]. Wang et al. used non-negative matrix factorization (NMF) on cancer clustering [16]. Among these methods, sparse methods have distinct advantages and catch the attention of more and more people. Until now, a large number of sparse methods were proposed. For instance, Wang et al. raised robust sparse PCA (SPCA) by using weighted elastic net [17]. A sparse PCA via low-rank approximations was proposed by Papailiopoulos et al. [18]. Witten et al. proposed a penalized matrix decomposition [19], which was used for differential expression analysis [20], [21]. In addition, many sparse methods have already been chosen to deal with the gene expression data. Liu et al. used the first principal component (PC) of SPCA for extracting plants core genes [22]. Yin et al. identified differential gene pathways with SPCA [23]. Zheng et al. discovered molecular pattern [24] based on PMD.

The sparse methods mentioned above were proverbially applied on gene expression data analysis and have made many remarkable achievements. But these methods are usually unsupervised while the category label of each sample in gene expression data has been already known. That is, the class information is neglected by these sparse methods when processing gene expression data. For example PMD was used to extract plants core genes by Liu et al. [20]. However, the category labels of samples are quite important for gene identification that many excellent gene selection algorithms were achieved by using the class information. For instance Guyon et al. proposed the Support Vector Machine-Recursive Feature Elimination (SVM-RFE) method to select genes for cancer classification [25]. SVM-RFE is a classic gene selection algorithm that it eliminate genes one by one by using Recursive Feature Elimination (RFE) and achieve a very good performance. Many extensions on SVM-RFE algorithm were proposed by scholars. Tang et al. developed a new two-stage SVM-RFE algorithm to gene selection for microarray expression data analysis [26]. Ding et al. improved the computational performance of SVM-RFE by eliminating chunks of features at a time with little effect on the quality of reduced feature set [27]. Since SVM-RFE was designed to handle the binary feature selection problems, it is not suitable for multiclass feature selection problems. In order to solve this issue, Zhou et al. proposed a family of four extensions to SVM-RFE to solve these problems [28]. Duan et al. computed the feature ranking score from a statistical analysis of weight vectors of multiple linear SVMs trained on subsamples of the original training data at each step [29].

Therefore, we bring in the class information by the total scatter matrix and put forward a novel method to improve the performance of PMD-based gene extraction method for identifying plants core genes responding to abiotic stresses. We called it a Class-Information-based Penalized Matrix Decomposition (CIPMD). The scheme of CIPMD is as follows. Firstly, the total scatter matrix is obtained based on samples of the gene expression data. Secondly, we decompose the total scatter matrix by using SVD, and construct a new data matrix via multiplying the left singular vectors by the singular values. Thirdly, the new data matrix is decomposed to get the sparse eigensamples by PMD. Finally, the core genes are identified according to the nonzero entries in eigensamples.

Our main contributions of this paper are as follows. On one hand, it is the first time that it puts forward the CIPMD method via integrating the class information into penalized matrix decomposition. On the other hand, to identify plants core genes responding to abiotic stresses, it provides plenty of experiments on simulation and real gene expression data.

The remainder of the paper is organized as follows. Section 2 describes the methodology of CIPMD. Section 3 presents the numerical experiments and discusses the results. The conclusion is shown in Section 4.

## Methodology

In this section, the class-information-based penalized matrix decomposition (CIPMD) method is introduced. Then, it is used to identify the core genes responding to the abiotic stresses.

### 2.1 The definition of CIPMD

In this subsection, we take the class information of samples into account and propose the CIPMD method to gain a better performance than PMD. At first, we bring in the class information via the total scatter matrix 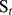. Then, a new data matrix is constructed by decomposing 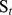. Finally, the new data matrix is decomposed by PMD. The following is our specific idea.

#### 2.1.1 The definition of scatter matrices

There exist many samples which contain different class labels in gene expression data. We take advantage of the class labels of samples via the total scatter matrix. For all the samples of all classes, we define three measures from the mathematical point of view. The first measure is named as a between-class scatter matrix (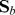) that is written as follows:
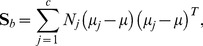
(1)where


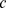
: the number of classes;


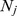
: the number of samples in class 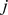;


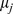
: the average value of class 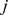;


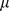
: the average value of all classes.

The second measure is named as a within-class scatter matrix (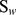) that is defined by
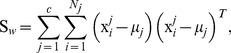
(2)where 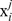 represents the 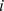-th sample of class 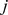.

The third measure is named as the total scatter matrix (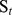) which is defined based on 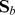 and 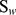. In order to minimize the within-class distance and maximize the between-class distance, the formula of 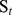 is given as follows:
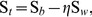
(3)where 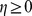 represents an adjustable parameter and gives a compromise between 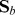 and 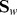.

The between-class and the within-class distances can be calculated by the trace of corresponding scatter matrices. In detail, the formulas are as follows:
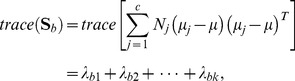
(4)

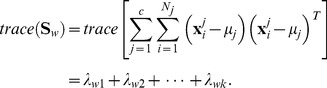
(5)


In the two formulas above, the separation of the samples between classes can be measured by the 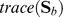 while the closeness of the samples within classes can be measured by the 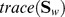. The parameter 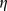 in eq. (3) is defined by [30]

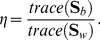
(6)


#### 2.1.2 Constructing the new data matrix 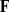


Due to the total scatter matrix 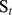 should be processed by PMD in a convenient way, so 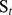 is preprocessed by matrix decomposition methods.

Firstly, the total scatter matrix 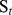 is decomposed by SVD, which can be written as follows:
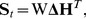
(7)where 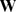 and 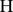 are orthogonal matrices, 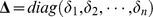 is the diagonal matrix which contains singular values, 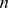 is the rank of the total scatter matrix 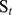.

Secondly, a new data matrix 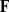 is constructed by
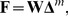
(8)where 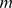 is the power of 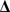. The suitable value of 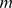 can be determined in Subsection 3.1.2 by using the simulation data.

Finally, the new data matrix 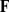 is decomposed by PMD.

In this way, the total scatter matrix 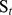 which contains large amounts of complex data is converted to the new data matrix 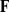 which is simple and easy to be processed.

#### 2.1.3 Penalized matrix decomposition (PMD)

In this subsection, we briefly introduce the PMD method proposed by Witten et al. [19]. Gene expression data always consist of 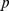 genes in 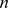 samples, in general, 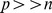. According to subsection 2.1.1 and subsection 2.1.2, the new data matrix 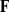 is obtained by calculating the original gene expression data. Therefore, we denote the gene expression data by the matrix 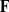 with size 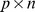. Without loss of generality, we let the row mean of 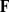 be zero. The matrix 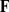 can be decomposed by SVD as follows:
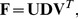
(9)where 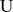 is a 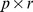 orthogonal matrix, 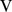 is an 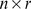 orthogonal matrix and 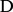 is a diagonal matrix. PMD can generalize this decomposition by imposing constraints on 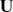 and/or 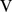. PMD can be represented as the following optimization problem:
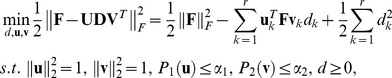
(10)where


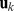
: the column 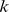 of 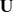;


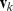
: the column 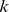 of 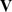;


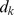
: the 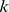-th diagonal element of 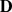;


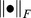
: the Frobenius norm;


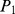
 and 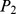: convex penalty functions that can adopt a various of forms [19].

When 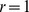, 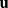 and 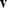 satisfying eq.(10) can also satisfy the optimization problem as follows [19]:
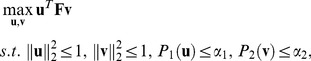
(11)and the 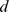 satisfying eq.(10) is 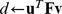. The objection function 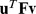 in eq.(11) is bilinear on 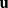 and 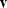, that is to say, when 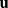 fixed, it is linear in 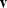, and vice versa. By choosing the appropriate 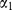 and 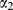, the solution to eq.(11) which is named as rank-one PMD satisfies eq.(10) [19].

The iterative algorithm for rank-one PMD is summarized as follows:

*Step1*. Initialize 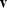 to have unit 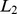-norm.

*Step2*. Iterate until convergence:

(a) 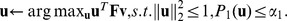


(b) 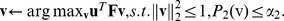


*Step3*. 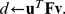


In order to obtain the rank-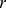 PMD, each time we use the residuals obtained by subtracting 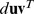 from 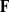 to maximize the eq.(11) repeatedly, i.e., 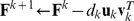. The specific algorithm of rank-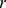 PMD can be found in [19]. In this research, we only impose the penalty on 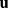, i.e. 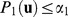, and do not consider 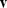 since core genes are identified according to 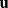. PMD can produce sparse vectors 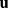 by choosing a suitable parameters 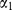.

### 2.2 Identifying core genes by CIPMD

The gene expression data are stocked as the matrix 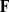 with size 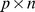, in which each row of 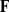 represents the transcriptional responses of a gene in all 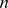 samples and each column of 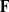 represents the expression level of a sample in all 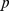 genes.

**Figure 1:**
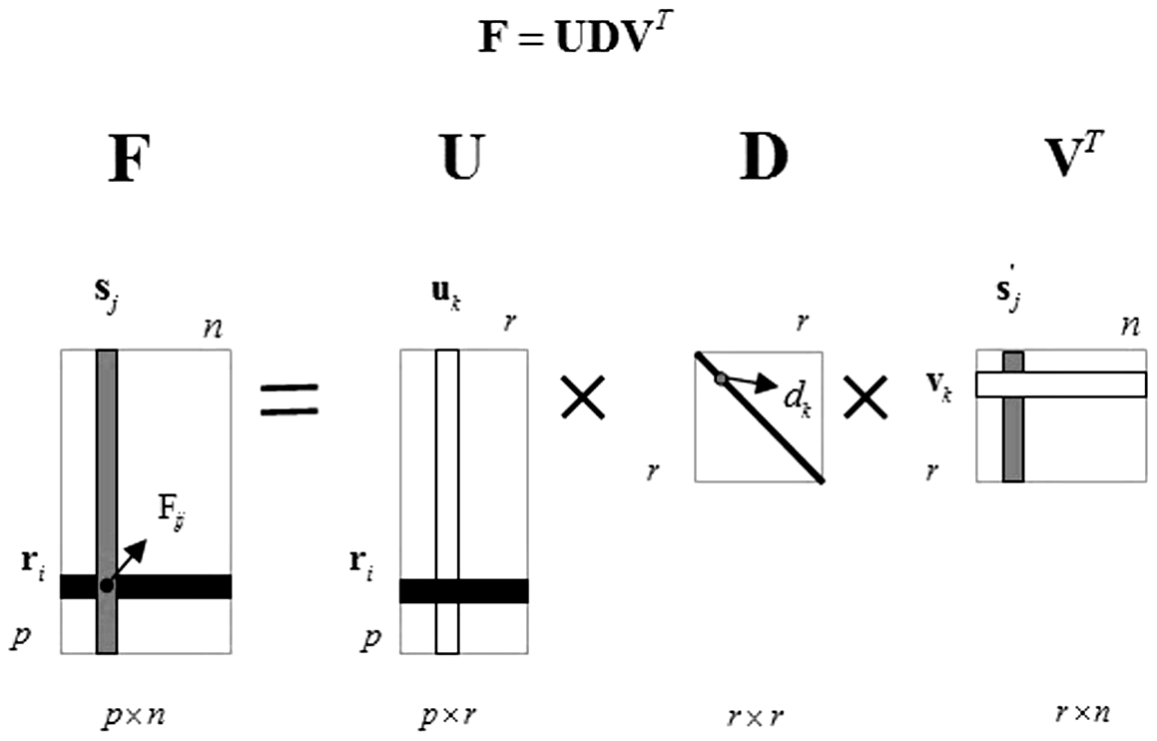
The graphical depiction of CIPMD. In this figure, the matrix 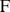 is decomposed into two bases matrices 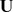, 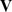 and a diagonal matrix 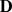.

According to subsection 2.1.3, the matrix 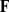 is decomposed into three matrices 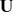, 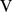 and 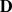 by PMD. The graphical depiction of CIPMD is shown in Figure 1. Following the convention in [31], we define 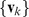 (columns of 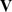) as eigenpatterns, 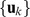 (columns of 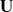) as eigensamples and 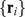 (rows of 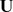) as eigengenes. As Figure 1 shows, the space of sample expression profiles 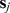 (a column of 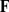) is spanned by 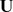 and the space of gene transcriptional responses 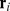 (a row of 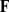) is spanned by 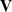.

Our goal is to identify the core genes from the gene expression data. Generally speaking, due to the complexity of 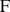, it is difficult to identify the core genes from 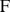 directly. So we must take measures to reduce the dimensionality of the gene expression data. As mentioned above, the space of sample expression profiles 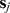 is spanned by 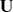 and 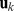 is a column of 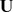, so we can select a subset of 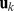 to represent 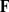. Then the eigengenes are identified from the eigensamples which have the features of gene expression data. These eigengenes are regarded as core genes responding to the abiotic stresses. The detail of how to identify the core genes from the sample expression profiles is shown in the following.

Firstly, the number of variables used to denote the sample expression profiles can be reduced by CIPMD. According to eq.(9), 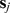 can be formulated as 
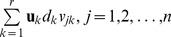
, where 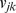 is the 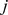-th element in 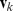. It shows that 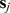 is a linear combination of 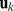. In Figure 1, 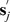 is the 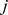-th column of 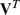, which includes the positional information of the 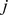-th sample. By using 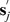, the expression profiles of samples can be acquired by 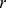 variables. However, the number of variables in sample expression profiles 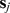 is 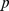 which is much larger than 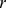. Therefore, the number of variables used to denote the sample expression profiles can generally be reduced by CIPMD.

Secondly, since the eigensamples 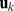 are used to reconstruct 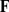, the sample expression profiles 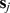 which contain the important information can be represented by the eigensamples 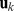.

Thirdly, the sparse 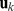 can be obtained by choosing the penalty function appropriately. According to the subsection 2.1.3, we can take penalty function 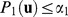. By choosing a suitable parameters 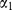, the sparse 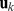 can be obtained.

Finally, the core genes responding to abiotic stresses are identified via the sparse 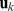. The features of samples in gene expression data can be represented by the nonzero entries in the sparse 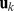. Therefore, the nonzero entries can be denoted as the core genes responding to abiotic stresses.

The whole scheme to identify the core genes can be summarized in the following:

Firstly, the total scatter matrix 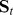 is obtained bases on the gene expression data 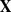.

Secondly, 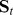 is decomposed into left singular vectors 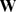, right singular vectors 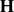 and a diagonal matrix 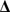 by using SVD, and a new data matrix 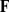 is constructed by multiplying 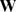 by 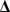.

Thirdly, PMD decomposes the data matrix 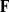 to obtain the sparse eigensamples 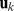.

Fourthly, the genes corresponding to nonzero entries in 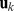 are identified as the core ones.

Finally, the core genes are checked by using Gene Ontology (GO) tool.

## Results and Discussion

In this section, we evaluate the CIPMD method by applying it to identify the core genes responding to abiotic stresses. Subsection 3.1 and 3.2 provide the results on simulation and real gene expression data sets, respectively. For comparison, the sparse principal component analysis (SPCA) [32], penalized matrix decomposition (PMD) [19] and support vector machine-recursive feature elimination (SVM-RFE) [25] methods are used to identify the features on simulation and real gene expression data sets. The LIBSVM that Chang et al. proposed [33] is used to implement SVM-RFE algorithm.

### 3.1 Results on simulation data

In this subsection, the simulation data are firstly introduced. Then, the parameters of SPCA, PMD and CIPMD are chosen appropriately. Since SVM-RFE method eliminate genes one by one by using Recursive Feature Elimination (RFE) and have no control-sparsity parameters, so we do not consider it in this subsection. Finally, the results on simulation data are shown.

#### 3.1.1 Data source

The simulation data are generated with 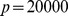 genes (roughly equal to the number of genes in real gene expression data) and 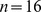 samples. In the two-class case, we assign 8 samples and 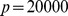
genes for each class. In the multi-class case, the 16 samples are divided equally into 4 classes.

The simulation data are in 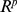 with 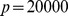 and generated as 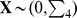. Let 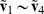 be four 20000-dimensional vectors, such that 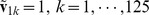, and 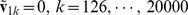; 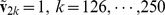, and 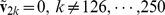; 
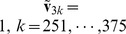
, and 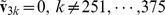; 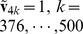, and 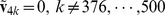. Let 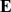 be a 20000-dimensional noise matrix, and 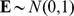. Then we add 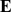 into 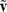 with different Signal-to-Noise Ratios (SNR). The preceding four eigenvectors of 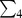 are normalized to be 
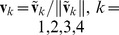
. And in order to make the first four eigenvectors dominate, we let the eigenvalues be 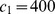, 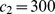, 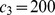, 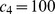 and 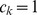 for 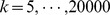. In this way, the simulation idea in [34] is applied to generate the simulation data.

#### 3.1.2 Parameters selection

In this subsection, the parameter 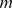 in eq.(8) is determined by the simulation experiment. Then the control-sparsity parameters of the three methods are selected appropriately.

**Figure 2:**
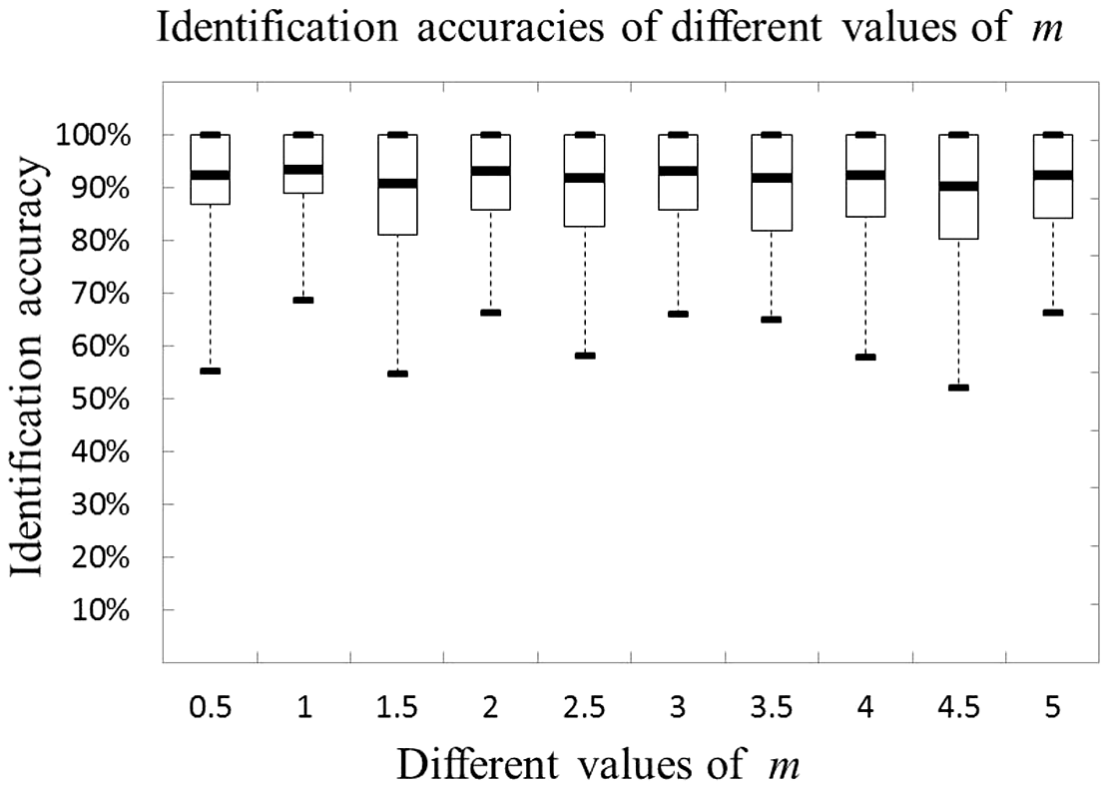
Accuracies of the CIPMD on simulation data set with different values of 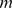.

- **The determination of parameter** 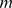 **:** For CIPMD, we need to determine the appropriate parameter 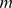 in eq.(8) to make our method optimal. We randomly generate the simulation data by iterating 100 times to test the performance of CIPMD with different values of 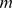. Figure 2 displays the performance of CIPMD with 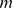 varying from 0.5 to 5. From this figure it can be seen that all the values of 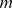 can get very high identification accuracies. The best result is achieved when 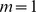, so we take 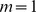 for CIPMD in the following experiments.
- **The selection of control-sparsity parameters:** Except for SVM-RFE, all the other three methods are sparse, whose control-sparsity parameters have a great influence on identification accuracy. The SPCA proposed by Journee et al. has an excellent performance both in computational speed and quality [32]. The parameter 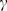 in SPCA is used to adjust the sparsity of PCs. According to the algorithm of CIPMD, the 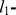
norm of 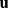 is taken as the penalty function, i.e. 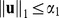. Since 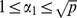, let 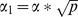, where 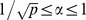. So we can obtain a sparse 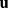 by choosing an appropriate 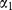. For simplicity, only one factor is used, that is, let 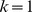.

For fair comparison, 500 genes are identified by using these methods with their own appropriate parameters. And the Signal-to-Noise Ratio (SNR) is set to be 0.1 when the simulation data are generated.

#### 3.1.3 Simulation results

**Figure 3:**
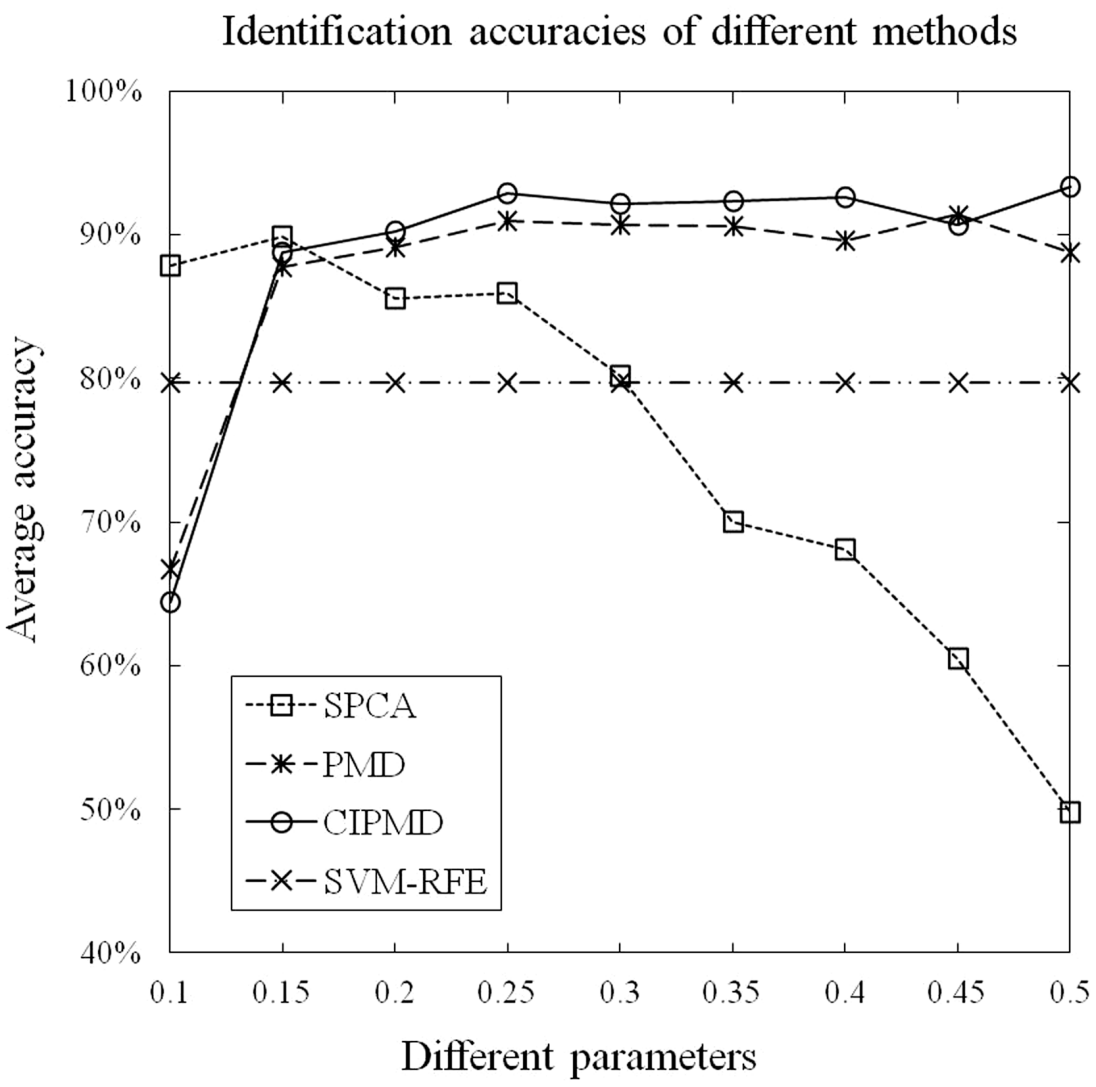
Accuracies of the four methods on simulation data with different paramaters in the case of two-class.

We randomly generate the simulation data by iterating 100 times to evaluate the performances of the four methods. The specific numerical values of identification accuracies of the four methods with different parameters are shown in supplementary file (Table S1). For the two-class case, the graphical depiction of the identification accuracies of these methods with different parameters is shown in Figure 3. From this figure, it can be seen that except for SVM-RFE, all the other three methods are sensitive to the control-sparsity parameters. The identification accuracies of SPCA are monotonically decreasing with the control-sparsity parameter when its value is greater than 0.15. On the contrary, the identification accuracies of PMD and CIPMD are monotonically increasing with the parameters when their values are smaller than 0.25. The identification accuracies of PMD and CIPMD are stabilized when the parameters are greater than 0.25. Moreover, all the four methods can obtain very high identification accuracies. Finally, our CIPMD has the highest accuracies among the four methods.

**Figure 4:**
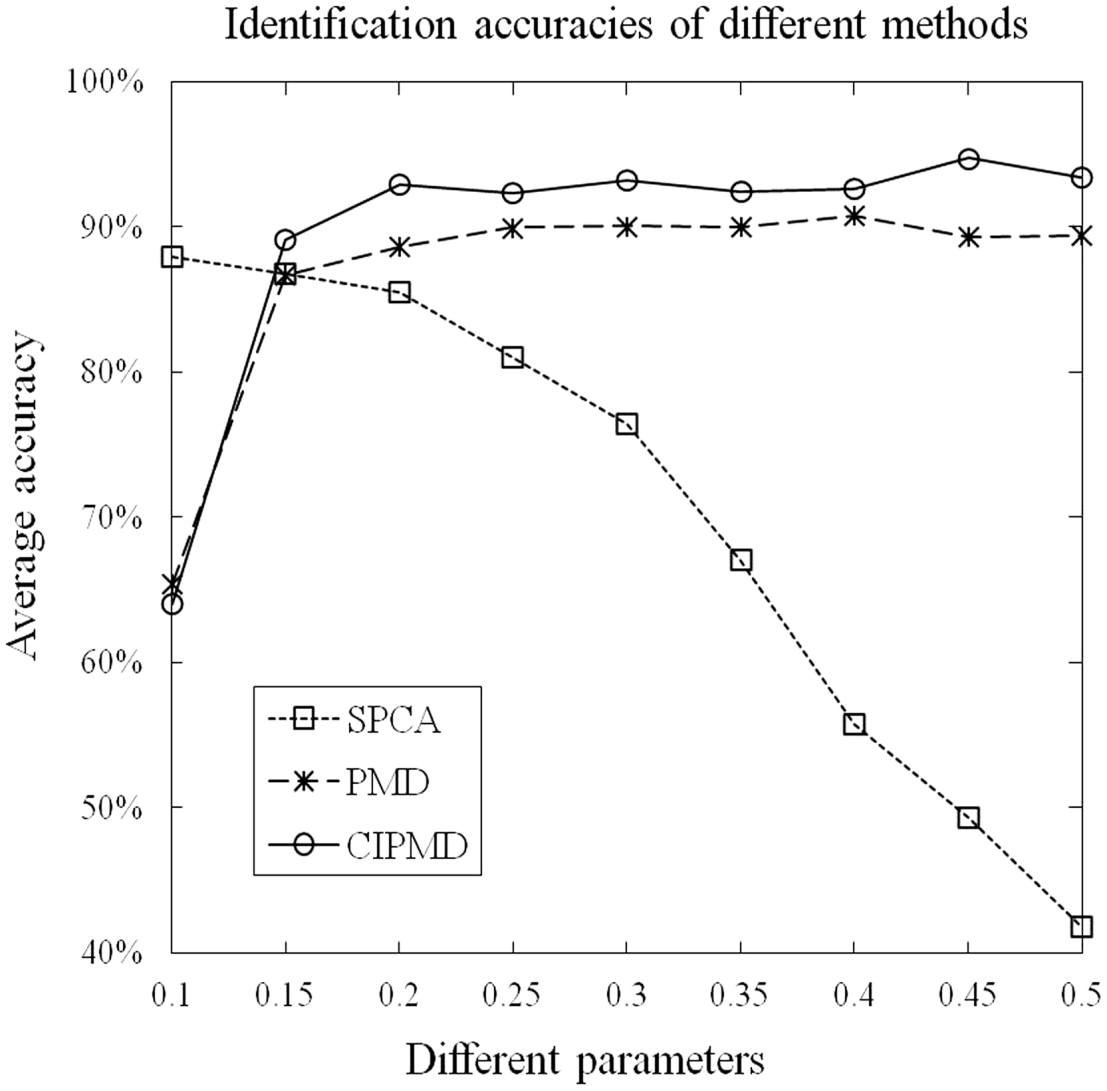
Accuracies of these methods on simulation data with different paramaters in the case of multi-class.

For the multi-class case, the graphical depiction of the identification accuracies of these methods with different parameters is shown in Figure 4. Since SVM-RFE was designed to deal with the binary gene selection problem, accordingly, it is not included in this part. From this figure, we can see that the identification accuracies of all the three methods can reach higher values. Similar to the two-class case, the identification accuracies of SPCA are monotonically decreasing with the increasing of control-sparsity parameter. When the parameters are greater than 0.2, the identification accuracies of CIPMD can reach the highest point and becomes stable. While the parameters are greater than 0.25, PMD reaches a plateau in terms of identification accuracy. Among the three methods, only the identification accuracies of CIPMD can reach more than 90%. Furthermore, except for the parameter is 0.1, CIPMD outperforms the other methods on identification accuracies with all parameters.

### 3.2 Results on gene expression data

The real gene expression data are introduced in subsection 3.2.1. Then, the gene ontology (GO) analysis is adopted to evaluate the performances of the four methods.

#### 3.2.1 Data source

The raw gene expression data include two classes: shoots and roots in each stress. The Affymetix CEL files are downloaded from NASCArrays [http://affy.arabidopsis.info/] [35], reference numbers are: control, NASCArrays-137; cold stress, NASCArrays-138; osmotic stress, NASCArrays-139; salt stress, NASCArrays-140; drought stress, NASCArrays-141; UV-B light stress, NASCArrays-144; and heat stress, NASCArrays-146. The number of samples in each stress type is listed in Table 1. There are 22810 genes in each sample. The arrays are adjusted by using the GC-RMA software by Wu et al. [36] to avoid the background of optional noise and normalized by using quantile normalization. The GC-RMA results are gathered in a matrix to be processed by SPCA, PMD, SVM-RFE and CIPMD.

**Table 1:** The number of each stress types in the raw data.

Stress Type	control	cold	drought	heat	osmotic	salt	UV-B
Number	8	6	7	8	6	6	7

Our method brings in the class information of samples based on the total scatter matrix. Therefore, in our experiments, two stress types of gene expression data are processed simultaneously.

#### 3.2.2 Gene Ontology (GO) analysis

Gene Ontology (GO) Term Enrichment tools can be used to describe genes in the input or query set and to help discover what functions the genes may have in common [37]. As a web-based tool, GOTermFinder can find the significant GO terms among a list of genes. Therefore, it offers some significant informations for the biological explanation of high-throughput experiments. The core genes responding to abiotic stresses identified by SPCA, PMD, SVM-RFE and CIPMD are checked by GOTermFinder which is publicly available at http://go.princeton.edu/cgi-bin/GOTermFinder [38]. Its threshold parameters are set as following: maximum P-value  = 0.01 and minimum number of gene products = 2. Here, only the main results of GO Term Enrichment are shown.

- **Terms responding to stimulus:** The numbers of genes responding to stimulus (GO:0050896), which is the ancestor of all the abiotic stresses, are identified by the four methods in shoot and root samples are listed in Table 2 and Table 3, respectively. The superior results are marked in bold type. From the two tables we can see that all these methods can identify genes with very high sample frequency and very low P-value.

**Table 2:** Response to stimulus (GO:0050896) in shoot samples.

Stress Type	SPCA		PMD		CIPMD		SVM-RFE	
	SF	PV	SF	PV	SF	PV	SF	PV
Cold	283 56.8%	1.33E-62	294 58.9%	3.89E-70	**329 65.9%**	**7.52E-98**	259 51.9%	8.6E-47
Drought	273 54.8%	7.04E-56	303 60.7%	8.84E-77	**338 67.7%**	**9.08E-106**	251 50.3%	4.07E-42
Heat	267 53.6%	5.00E-52	220 44.0%	5.84E-26	**330 66.0%**	**2.39E-98**	271 54.3%	2.93E-54
Osmotic	264 52.9%	6.24E-50	296 59.2%	2.70E-71	**322 64.5%**	**6.15E-92**	243 48.6%	1.84E-37
Salt	264 52.8%	1.06E-49	**258 51.8%**	**1.68E-46**	309 61.9%	2.00E-81	256 51.2%	8.25E-45
UV-B	334 67.1%	1.36E-102	361 72.3%	1.8E-127	**335 67.3%**	**1.81E-103**	243 48.6%	1.92E-37

In this table, the response to stimulus on core genes are shown, whose background frequency in TAIR is 6619/30324 (21.8%), where 6619/30324 represents having 6619 genes response to stimulus in whole 30324 genes. SF and PV represent the sample frequency and P-value, respectively. The sample frequency, e.g. 283, represents the method identifies 500 genes, in which there are 283 genes responding to stimulus.

**Table 3:** Response to stimulus (GO:0050896) in root samples.

Stress Type	SPCA		PMD		CIPMD		SVM-RFE	
	SF	PV	SF	PV	SF	PV	SF	PV
Cold	282 56.6%	6.57E-62	291 58.2%	1.07E-67	**337 67.5%**	**6.92E-105**	267 53.7%	3.49E-52
Drought	289 57.8%	2.91E-66	287 57.4%	7.60E-65	**333 66.6%**	**5.54E-101**	273 54.8%	8.59E-56
Heat	199 39.9%	3.46E-17	205 41.1%	1.34E-19	**337 67.5%**	**7.27E-105**	283 56.6%	5.63E-62
Osmotic	238 47.6%	7.62E-35	223 44.6%	2.03E-27	**302 60.6%**	**2.54E-76**	276 55.2%	2.86E-57
Salt	238 47.6%	7.31E-35	313 62.6%	2.75E-84	**295 59.0%**	**1.31E-70**	264 52.9%	7.69E-50
UV-B	211 42.2%	5.55E-22	**243 48.6%**	**1.53E-37**	326 65.2%	6.85E-95	268 53.7%	2.37E-52

As Table 2 listed, in shoot samples, only in UV-B light stress data set, CIPMD method is dominated by PMD. For other stresses data sets, CIPMD outperforms the SPCA, PMD and SVM-RFE. As Table 3 listed, in root samples, CIPMD performs better than the other three methods in all the stresses data sets except the salt stress. In salt stress data set, PMD method is superior to our method.

**Figure 5:**
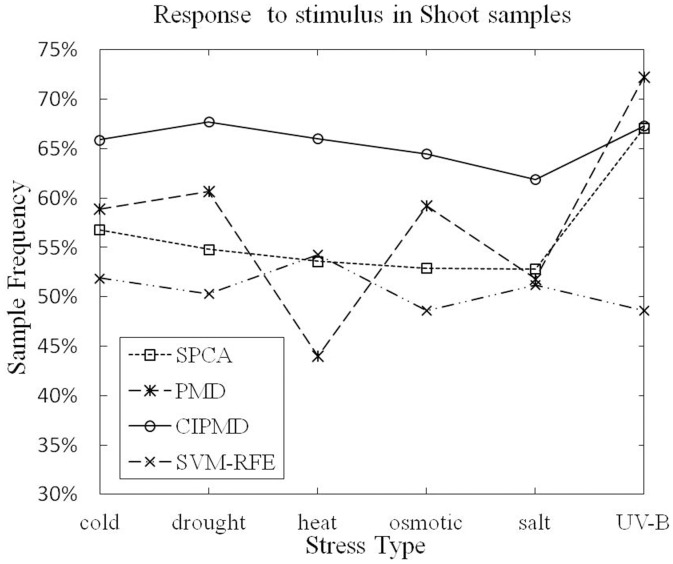
Response to stimulus (GO:0050896) in shoot samples.

The sample frequencies of the six different stresses response to stimulus in shoot and root samples are shown in Figure 4 and Figure 5, respectively.

**Figure 6:**
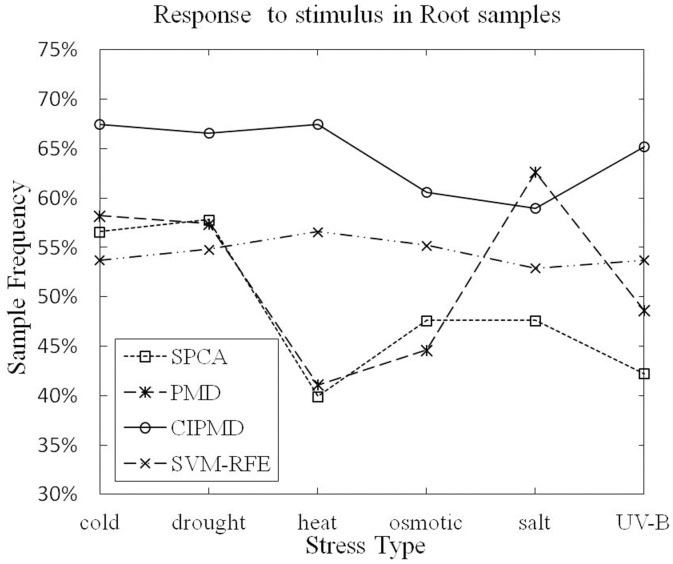
Response to stimulus (GO:0050896) in root samples.

From Figure 5, it can be seen that PMD has a higher data point on UV-B light stress data set than SPCA, SVM-RFE and CIPMD. However, CIPMD method is superior to PMD, SPCA and SVM-RFE in the remaining five stresses data sets of shoot samples. Figure 6 shows that only in salt stress data set, CIPMD has a lower data point than PMD. CIPMD method outperforms the other three methods in a large degree (especially in heat stress data set) in other five stresses data sets of root samples. From the two figures we can also find that SVM-RFE and CIPMD give more stable results in six different stresses data sets than PMD and SPCA methods whose results fluctuate up and down in greatly amplitudes.

PMD outperforms the proposed method in some case of the experiment, e.g. the UV-B light stress data set in shoot samples and the salt stress data set in root samples, the most likely reason is that the different distributions of data lead to the different performances between methods. This problem also exists in elsewhere, for example Zheng et al. proposed a gene selection method based on Robust Principal Component Analysis (RPCA) to select plants characteristic genes, in their experiments, the number of genes responding to abiotic stimulus (GO:0009628) is selected by three methods in root samples, the performance of RPCA is equal to PMD only in UV-B stress data set, in other data sets, RPCA method is superior to the others [39].

- **Terms responding to stress:** Table 4 and Table 5 list the gene numbers and P-value of response to stress (GO:0006950) in shoot and root samples, respectively. The superior results are marked in bold type.

**Table 4:** Response to stress (GO:0006950) in shoot samples.

Stress Type	SPCA		PMD		CIPMD		SVM-RFE	
	SF	PV	SF	PV	SF	PV	SF	PV
Cold	219 44.0%	1.47E-61	213 42.7%	4.44E-57	243 48.7%	6.84E-80	204 40.9%	1.07E-50
Drought	198 39.8%	5.05E-47	246 49.3%	2.47E-82	255 51.1%	5.89E-90	201 40.3%	1.02E-48
Heat	187 37.6%	4.49E-40	174 34.8%	3.51E-32	264 52.8%	1.51E-97	225 45.1%	1.21E-65
Osmotic	192 38.5%	4.96E-43	227 45.4%	4.12E-67	246 49.3%	2.30E-82	183 36.6%	2.88E-37
Salt	169 33.8%	1.85E-29	176 35.3%	1.34E-33	236 47.3%	2.90E-74	202 40.4%	3.32E-49
UV-B	249 50.0%	4.18E-85	295 59.1%	3.3E-127	277 55.5%	1.06E-109	186 37.2%	4.81E-39

In this table, the response to stress on core genes are shown, whose background frequency in TAIR is 4028/30324 (13.3%), where 4028/30324 represents having 4028 genes to response to stress in whole 30324.

**Table 5:** Response to stress (GO:0006950) in root samples.

Stress Type	SPCA		PMD		CIPMD		SVM-RFE	
	SF	PV	SF	PV	SF	PV	SF	PV
Cold	223 44.8%	1.66E-64	233 46.6%	9.92E-72	**264 52.9%**	**7.64E-98**	218 43.9%	7.14E-61
Drought	231 46.2%	3.60E-70	222 44.4%	2.27E-63	**279 55.8%**	**2.50E-111**	225 45.2%	7.69E-66
Heat	152 30.5%	5.73E-21	169 33.9%	1.39E-29	**277 55.5%**	**1.03E-109**	242 48.4%	1.11E-78
Osmotic	172 34.4%	4.39E-31	160 32.0%	8.07E-25	**234 46.8%**	**1.78E-72**	227 45.4%	6.15E-67
Salt	178 35.6%	1.79E-34	**246 49.2%**	**3.88E-82**	232 46.4%	5.58E-71	218 43.7%	1.76E-60
UV-B	153 30.6%	2.26E-21	165 33.0%	2.34E-27	**262 52.4%**	**9.89E-96**	222 44.5%	2.04E-63

As Table 4 listed, in shoot samples, CIPMD is superior to the other three methods in all the data sets except UV-B light stress. PMD suppresses our method only in the UV-B light stress data set. As Table 5 listed, in root samples, CIPMD is dominated by PMD only in salt-stress data set. CIPMD outperforms our competitive methods in other five stresses data sets.

**Figure 7:**
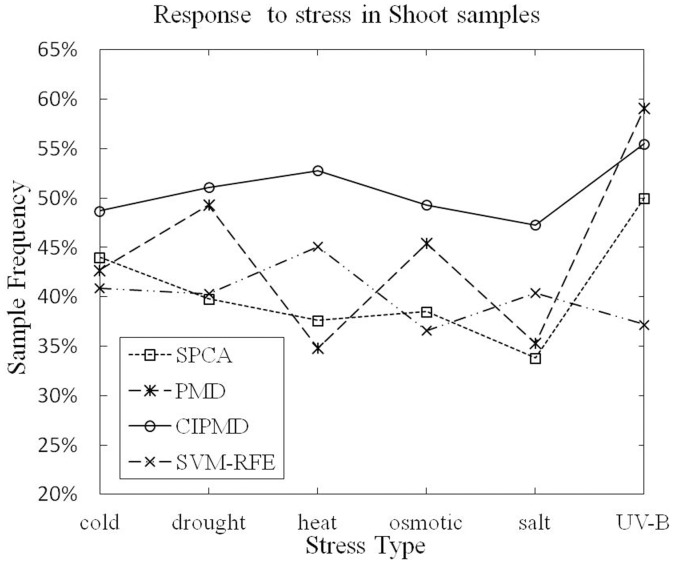
Response to stress (GO:0006950) in shoot samples.

**Figure 8:**
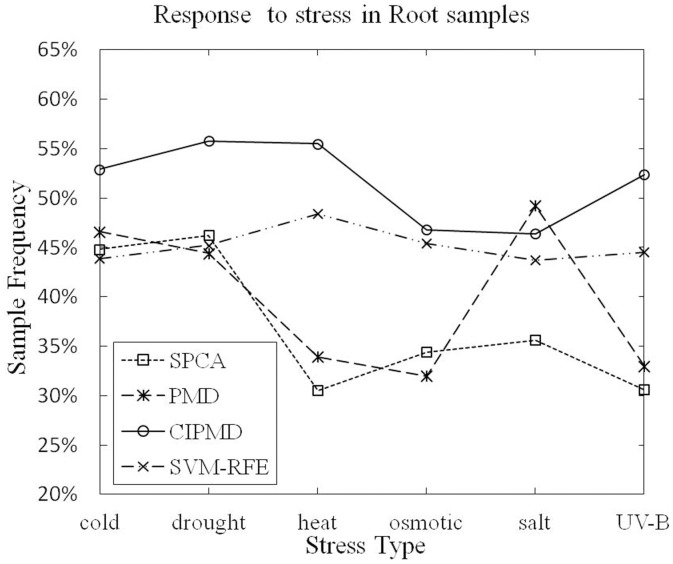
Response to stress (GO:0006950) in root samples.

The sample frequencies of response to stress in shoot and root samples are shown in Figure 7 and Figure 8, respectively.

Figure 7 shows that CIPMD method owns a lower data point in UV-B light stress data set than PMD. But in the rest five stresses data sets of shoot samples, our CIPMD is superior to the other methods. From Figure 8, it can be proved that PMD has a data point performing better than CIPMD only in salt stress data set. CIPMD method surpasses PMD and SPCA in a large extent (especially in heat stress data set) in other data sets of root samples. CIPMD outperforms SVM-RFE in all the data sets with six different stresses. Besides, both in shoot and root samples, CIPMD and SVM-RFE present more stable results than PMD and SPCA in six different stresses data sets.

- **Core genes responding to the stresses:** The data of the drought stress in root samples and heat stress in shoot samples are analyzed to evaluate the core genes identified by our method closely related to the stresses.

For drought stress in root samples, Table 6 gives the sample frequency and P-value of response to water deprivation (GO: 0009415). The background sample frequency of response to water deprivation (GO: 0009415) in root samples is 1.4% (421/30324). As Table 6 listed, the superior results of the three methods are shown in bold type. Obviously, CIPMD can identify more genes than the other three methods.

**Table 6:** The numbers of response to water deprivation (GO:0009415) in root samples.

Stress Type	SPCA		PMD		CIPMD		SVM-RFE	
	SF	PV	SF	PV	SF	PV	SF	PV
Drought	44 8.8%	1.73E-19	51 10.2%	6.21E-26	**69 13.8%**	**7.8E-45**	45 9.0%	2.53E-20

Moreover, we compare the genes identified by CIPMD with the ones identified by PMD, SPCA and SVM-RFE to verify the core genes extracted by our method closely related to abiotic stresses. Table 7 lists different genes identified by CIPMD and ignored by other three methods in the first column. The column of *Response to* represents what stresses the genes response to, and the column of *Reference* denotes the searching results that the authors have already confirmed in their literatures. As Table 7 listed, all the 27 genes selected by CIPMD and neglected by PMD, SPCA and SVM-RFE can be searched in literatures. And all these core genes are indeed closely related to drought stress. Furthermore, some of the genes are also related to cold, osmotic and salt stresses.

**Table 7:** References about core genes responding to water deprivation in root samples.

Gene name	Response to	References
At2g33380	Drought, cold	Heyndrickx et al. (2012) [40]
At4g34390	Drought	Heyndrickx et al. (2012) [40]
At5g62470	Drought	Seo et al. (2009) [41]
At3g14050	Drought	Heyndrickx et al. (2012) [40]
At3g11820	Drought, cold	Heyndrickx et al. (2012) [40]
At3g19970	Drought	Heyndrickx et al. (2012) [40]
At5g54490	Drought	Heyndrickx et al. (2012) [40]
At5g27420	Drought	Heyndrickx et al. (2012) [40]
At4g24960	Drought, cold	Chen et al. (2002) [42]
At2g30550	Drought	Heyndrickx et al. (2012) [40]
At3g30775	Drought	Sharma et al. (2011) [43]
At3g63060	Drought, salt, osmotic	Koops et al. (2011) [44]
At3g09940	Drought	Vadassery et al. (2009) [45]
At4g21440	Drought	Heyndrickx et al. (2012) [40]
At1g73480	Drought, cold	Heyndrickx et al. (2012) [40]
At5g67340	Drought, cold	Heyndrickx et al. (2012) [40]
At4g17500	Drought	Heyndrickx et al. (2012) [40]
At2g17840	Drought, cold	Kiyosue et al. (1994) [46]
At3g52400	Drought, cold	Fujita et al. (2004) [47]
At4g05100	Drought	Heyndrickx et al. (2012) [40]
At5g24590	Drought	Heyndrickx et al. (2012) [40]
At5g67300	Drought	Huang et al. (2008) [48]
At5g40390	Drought	Maruyama et al. (2009) [49]
At3g19580	Drought	Sakamoto et al. (2000) [50]
At5g45340	Drought	Umezawa et al. (2006) [51]
At1g22190	Drought, cold, osmotic	Rea et al. (2011) [52]
At3g57530	Drought, cold	Heyndrickx et al. (2012) [40]

For heat stress in shoot samples, Table 8 lists the sample frequency and P-value of response to heat (GO: 0009480). The background sample frequency of response to heat (GO: 0009480) in shoot samples is 1.0% (298/30324). In Table 8, the superior results of the four methods are marked in bold type. Wherein SVM-RFE cannot identify effective genes response to heat. It can be seen clearly that CIPMD method can identify more genes than the other methods.

**Table 8:** The numbers of response to heat (GO:0009408) in shoot samples.

Stress Type	SPCA	PMD	CIPMD	SVM-RFE
	SF	PV	SF	PV	SF	PV	SF	PV
Heat	41 8.2%	1.13E-22	77 15.4%	2.37E-66	**97 19.4%**	**9.47E-96**	None	None

In detail, we compare the genes identified by CIPMD with the ones identified by using PMD, SPCA and SVM-RFE. There are 20 different core genes identified by our method and neglected by PMD, SPCA and SVM-RFE. Among these 20 genes, 14 genes responding to heat have been confirmed in literatures. We show the verified results of the 14 genes in Table 9. The remaining genes of the 20 genes are involved in heat acclimation (GO: 0010286) which is the children of response to heat (GO: 0009408). The affirmed results in literatures of the 6 genes are listed in Table 10. From the verifications, it is obvious that all the 20 genes identified by CIPMD and ignored by PMD, SPCA and SVM-RFE are closely related with heat stress.

**Table 9:** References about core genes responding to heat in shoot samples.

Gene name	Response to	References
At2g43630	Heat	Heyndrickx et al. (2012) [40]
At1g14360	Heat	Heyndrickx et al. (2012) [40]
At5g28540	Heat	Koizumi et al. (1996) [53]
At2g20940	Heat	Heyndrickx et al. (2012) [40]
At4g29520	Heat	Heyndrickx et al. (2012) [40]
At4g29330	Heat	Heyndrickx et al. (2012) [40]
At5g22060	Heat	Heyndrickx et al. (2012) [40]
At1g04980	Heat	Heyndrickx et al. (2012) [40]
At4g00940	Heat	Heyndrickx et al. (2012) [40]
At3g10800	Heat	Gao et al. (2008) [54]
At4g16660	Heat	Heyndrickx et al. (2012) [40]
At1g07410	Heat	Heyndrickx et al. (2012) [40]
At5g56030	Heat	Takahashi et al (1992) [55]
At2g02810	Heat	Heyndrickx et al. (2012) [40]

**Table 10:** Reference about core genes involved in heat acclimation in shoot samples.

Gene name	Response to	References
At5g38895	Heat	Heyndrickx et al. (2012) [40]
At1g77000	Heat	Lim et al (2006) [56]
At4g02550	Heat	Heyndrickx et al. (2012) [40]
At3g50970	Heat, drought	Heyndrickx et al. (2012) [40]
At1g13080	Heat	Lim et al (2006) [56]
At4g11220	Heat	Heyndrickx et al. (2012) [40]

## Conclusion

In this study, we proposed a novel Class-Information-based Penalized Matrix Decomposition method for identifying core genes. Our method can achieve a better identification capacity by bringing in the class information of samples based on the total scatter matrix. By integrating matrix decomposition and the PMD method, our method is appropriate to analyze the gene expression data. A large number of experiments on simulation and real gene expression data demonstrate that our CIPMD method outperforms both PMD, SPCA and SVM-RFE. Thus, our approach is effective to identify plants core genes responding to abiotic stresses.

In the future, we will focus on the biological interpretation of the core genes.

## Supporting Information

Table S1 **The identification accuracies of the four methods with different parameters.**
(XLSX)
